# Exploitation of the Antibacterial Properties of Photoactivated Curcumin as ‘Green’ Tool for Food Preservation

**DOI:** 10.3390/ijms23052600

**Published:** 2022-02-26

**Authors:** Zunaira Munir, Giuliana Banche, Lorenza Cavallo, Narcisa Mandras, Janira Roana, Raffaele Pertusio, Eleonora Ficiarà, Roberta Cavalli, Caterina Guiot

**Affiliations:** 1 Department of Neurosciences, University of Turin, 10124 Turin, Italy; zunaira.munir@unito.it (Z.M.); raffaele.pertusio@unito.it (R.P.); caterina.guiot@unito.it (C.G.); 2 Bacteriology and Mycology Laboratory, Department of Public Health and Pediatric Science, University of Torino, Via Santena 9, 10126 Turin, Italy; giuliana.banche@unito.it (G.B.); lorenza.cavallo@unito.it (L.C.); janira.roana@unito.it (J.R.); 3 Department of Drug Science and Technology, University of Turin, 10125 Turin, Italy; roberta.cavalli@unito.it

**Keywords:** curcumin, photoactivation, food preservation, antibacterial properties

## Abstract

In the search for non-chemical and green methods to counteract the bacterial contamination of foods, the use of natural substances with antimicrobial properties and light irradiation at proper light waves has been extensively investigated. In particular, the combination of both techniques, called photodynamic inactivation (PDI), is based on the fact that some natural substances act as photosensitizers, i.e., produce bioactive effects under irradiation. Notably, curcumin is a potent natural antibacterial and effective photosensitizer that is able to induce photodynamic activation in the visible light range (specifically for blue light). Some practical applications have been investigated with particular reference to food preservation from bacterial contaminants.

## 1. Introduction

The concept of food preservation has its origins in ancient times when our ancestors tried to find methods to keep food fresh and edible (e.g., sun-drying, salting and pasteurization). The possibility of optimally preserving food for long periods is increasingly relevant: if food products are not subjected to treatments that ensure their proper preservation and decontamination by microorganisms, they are subject to more rapid deterioration and can be responsible for serious foodborne infections [1]. Classical food preservation techniques rely on physical and physicochemical action treatments, such as heat, freezing, radiation, and ultrasound [2]. However, some disadvantages have been discovered in these procedures, such as reductions in food volume and texture and losses of nutrients and organic properties, leading to huge overall losses in food products [3].

Therefore, scientific and technological researchers have been increasingly interested in the study of new antimicrobial materials and new food sanitization methods that can extend shelf-lives while ensuring their safety for humans. Thanks to their broad spectrum of activity, natural antimicrobials of plant origin are promising candidates to prevent food contamination by harmful microorganisms.

Light irradiation is another interesting ‘green’ approach. It has been extensively used as a food decontaminant via X-rays, ultrasound, UV, and visible light, among others. A detailed description can be found in the work of Cossu et al. [4].

More recently, light-emitting diodes (LED) in the range of 400–460 nm were found to very effective against bacteria. The mechanism that is assumed to play a major role in their effectiveness is the excitation of endogenous porphyrins that catalyze the production of intracellular reactive oxygen species (ROS), inducing cell oxidation and death [5]. The different responses of Gram-positive and -negative bacteria could be therefore explained by the different contents of porphyrins. To produce effective results in some food matrices, especially fruits and juices, LED exposition should last several hours unless photosensitizers (PS) are used. PS are non-toxic molecules that interact with irradiating light by generating radical species responsible for massive and aggressive oxidation, finally resulting in cell lysis and death. This effect is enhanced by the presence of molecular oxygen [6].

Both natural antimicrobial properties and PS are shared by curcumin, a natural component of *Curcuma longa*, and its antimicrobial properties against a wide variety of microorganisms, including bacteria and fungi, have been demonstrated in vitro [7]. Curcumin, in addition to exhibiting remarkable antioxidant, anti-inflammatory, and anticancer activities [8], benefits from a high availability on the market, safety even at high doses, and low cost [9]. In addition, due to its ability to emit fluorescence, its antimicrobial efficacy can be enhanced by photoactivation.

Curcumin is one of the strongest natural PS, with a light absorption peak between 400 and 500 nm suitable for excitation by blue light. Moreover, the irradiation breaks down the curcumin molecule, thus limiting its presence in the decontaminated final product [10].

However, the potential of this natural extract is limited by its poor water solubility and instability at physiological pH, leading to poor absorption and rapid degradation by hydrolysis and molecular fragmentation [7].

## 2. Principal Microbial Contaminant of Food

Foods responsible for illness, hospitalization, and death include meat, poultry, dairy, fruit, vegetables, seafood, grains, and nuts. Pathogenic microorganisms, particularly bacteria or fungi, can contaminate food at different stages such as during production and processing, as well as during the storage and transportation of the final product. In the course of replication, some contaminating bacteria can produce virulence factors and harmful substances such as toxins, which are involved in the pathogenesis of various infections [11].

The bacterium responsible for most foodborne infections is *Staphylococcus aureus*, a Gram-positive, commensal, and opportunistic pathogenic microorganism that can cause a wide spectrum of infections. *S. aureus* is known to be resistant to several chemical and physical factors: in fact, it grows in environments with a temperature ranging from 7 to 48.5 °C (optimal: 30–37 °C), a pH from 4.2 to 9.3 (optimal: 7–7.5), and a sodium chloride (NaCl) concentration of up to 15%.

Another etiologic agent of foodborne illness is *Escherichia coli*, specifically the enterohemorrhagic serogroup O157:H7. This Gram-negative species harmlessly colonizes the gastrointestinal tracts of humans and animals. However, there are strains that have become pathogenic because they have acquired virulence factors through plasmids, transposons, bacteriophages, and/or islands of pathogenicity. Unfortunately, there is no specific treatment for *E. coli* O157:H7 infection, and the use of antibiotics may be discouraged; consequently, the most widely used approach is supportive therapy to limit symptoms, halt disease evolution, and prevent systemic inconveniences [12]. 

Another category of bacteria that has become very important in recent times is represented by enterococci, which are very resistant by nature and able to survive, even for long periods of time, in a wide range of hostile environmental conditions. Enterococci are common commensals of the gastrointestinal tract, play important roles in food maturation and the development of specific flavors (such as in various cheeses), and can cause spoilage in some meats. However, these Gram-positive bacteria are the etiological agents of several nosocomial infections and can occur as food contaminants. Among them, the predominant species is *Enterococcus faecalis***,** a Gram-positive bacterium often associated with several pathological conditions including urinary tract infections, bacteremia, meningitis, wound infections, dental diseases, and neonatal infections [13].

Further details on their characteristics, including how their infections suffer from the development of antibiotic resistance (which is one of the most severe and harmful modern-day health problems) are provided in Table 1. 

In addition to bacterial contamination, fungal and viral contamination can seriously reduce food conservation and threaten human health. Fungal food spoilage plays a pivotal role in the deterioration of food and feed systems, and some fungi are also able to produce toxic compounds for humans and animals. The mycotoxins produced by fungi can cause serious health hazards, including Kashin–Beck disease and cancerogenic, immunotoxic, teratogenic, neurotoxic, nephrotoxic, and hepatotoxic effects. Additionally, fungal spoilage/pathogens can cause losses of the marketable quality and hygiene of foodstuffs, resulting in major economic problems throughout the world [22,23,24,25].

A recent review focused on mycotoxins produced mainly by *Aspergillus*, *Fusarium*, *Penicillium* and *Alternaria* fungi that affect cereals and spices, as well as on the need for safe decontaminants [26,27]. According to Liu et al., most of these mycotoxins are stable, and non-thermal approaches are required to counteract post-harvest fungi contamination [28]. Fresh food is often contaminated by viruses, such as noroviruses and hepatitis. A review of Yeargin and Gibson focused on viruses contaminating leafy greens, red fruits, and mollusks that require specific surveillance [29]. The current prevention strategies were extensively described in Shukla et al. [30].

The pathogenic bacteria described so far are not the only ones able to contaminate food. In fact, various food products can be subject to contamination by many other microorganisms responsible for infections. For this reason, in recent times, scientific and technological research has been increasingly focused on the study of new antimicrobial materials and new food sanitization methods in order to reduce contamination by microorganisms and lengthen the shelf-lives of food products while ensuring their safety to humans. 

There have been few studies of foodborne pathogens in the literature, but the consequences of exposure through food can be severe and a considerable number of people suffer from them every year [31].

## 3. Curcumin: Chemical Characterization

Since ancient times, turmeric/curcumin has been used as a spice, especially in India and other Asian countries. In addition, it is commonly used for medicinal purposes, especially to treat inflammatory conditions [32]. Curcumin (C_21_H_20_O_6_; 1,7-bis-4-hydroxy-3-methoxyphenyl-1,6-heptadiene-3,5-dione, molecular weight = 368,38 g/M) is a polyphenolic compound derived from the rhizomes of *Curcuma longa* L.

Regarding its chemical structure, curcumin contains two highly polar aromatic rings connected by means of a seven-carbon aliphatic chain and two α, β-unsaturated carbonyl groups (β-diketone). This chain is responsible for the hydrophobic nature of curcumin, and it additionally occurs with two tautomeric conformations: ketone and enol. The presence of these two forms is due to the intramolecular transfer of hydrogen atoms throughout the β-diketone molecule. The enol form is energetically more stable in solution and the solid state than the ketone form due to strong intramolecular hydrogen bonding. The tautomeric ketone form prevails in acidic/neutral aqueous solutions and cell membranes, whereas the enolic form is predominantly found in alkaline environments. Several factors determine the relative contribution of tautomeric forms in solution, including solvent polarity, temperature, and aromatic ring substitution [7]. According to some advanced nuclear magnetic resonance (NMR) studies, the dominance of the enolic form of curcumin in most organic solvents has been confirmed [33]. However, the likelihood of the re-equilibration of the two forms under specific conditions (e.g., in an acidic environment) is not excluded. Intramolecular hydrogen transfer determines the conversion of curcumin to the enolic form in nonpolar, aprotic solvents (such as deuterated chloroform), whereas the breakdown of an intramolecular hydrogen bond with an intermolecular hydrogen bond in protic solvents (such as methanol) leads to the conversion of curcumin in the enolic form to the ketonic form [7]. This step is critical because the poor solubility of curcumin in aqueous solutions could be due to the presence of these inter- and intra-hydrogen bonds [34]. Generally, the presence of diverse functional groups in a curcumin molecule (such as β-dicotyl groups, carbon–carbon double bonds, and phenyl rings with various hydroxyl and methoxyl substituents) determines its various biological activities. For example, curcumin exhibits a high antioxidant capacity, as it is able to neutralize several reactive species, such as superoxide anions, nitrogen dioxide radicals, and ROS [35]. The neutralization of these substances serves to prevent damage to bio-macromolecules. However, curcumin has both antioxidant and pro-oxidant activity—in fact, it is able to produce cytotoxic reactive oxygen species when exposed to light [7]. The oxidation mechanism and antioxidant capacity of curcumin are determined by the number of hydroxyl groups and their position in the aromatic ring; they also make the enolic form more susceptible to oxidation than the ketone form. 

Though curcumin has been considered to be a promising antibacterial drug with therapeutic potential, the major drawbacks of taking curcumin alone appear to be mostly due to its poor absorption, quick metabolism, and rapid elimination [36]. Curcumin bioavailability has been a source of controversy in recent years and has been improved by combining certain compounds to generate a curcumin complex. Piperine, for example, increases the bioavailability of curcumin by 20 times [37]. Curcumin’s water dispersibility, chemical stability, bioaccessibility, absorption, and overall bioavailability can all be improved with micelles and microemulsions. For example, curcumin microemulsions made from food-grade components such Tween 20, lecithin, vitamin E, and ethanol were found to improve curcumin water dispersibility by 1000–10,000 times [38]. Additionally, curcumin-loaded nanoemulsions can be successfully incorporated into commercial food products such as milk [39], as it was found that the addition of emulsified curcumin to milk reduced lipid oxidation.

The chemical structure of curcumin is also responsible for its ability to absorb light. This property is mainly due to the presence of alternating single and double bonds in the carbon chain. Curcumin possesses a broad absorption spectrum, especially in the visible region (420 nm), and it is capable of absorbing light up to a wavelength of about 430 nm. However, the transition to the ketone form of curcumin results in maximum absorption in the UV region (approximately 389 nm) [7].

## 4. Mechanisms of Antimicrobial Action of Curcumin

Curcumin exhibits antimicrobial activity, particularly against bacteria, and several studies have been conducted to demonstrate this antibacterial activity. For example, in one study, the antibacterial activity of turmeric oil against *Bacillus subtilis*, *B. coagulans*, *B. cereus*, *S. aureus*, *E. coli*, and *Pseudomonas aeruginosa* was tested and verified [40]. Curcumin has also shown inhibitory activity on methicillin-resistant *S. aureus* (MRSA) strains [41]. The antibacterial activity of curcumin comes from its ability to inhibit the assembly of the FtsZ protein in the Z loop, resulting in the blockage of bacterial cell division [42]. In addition, curcumin possesses anti-biofilm activity against bacteria. This was demonstrated in a study conducted on plant and animal models (*Arabidopsis thaliana* and *Caenorhabditis elegans*) infected with *P. aeruginosa*, in which the ability of curcumin to inhibit the production of genes involved in the early stages of biofilm formation was demonstrated [43].

**Figure 1:**
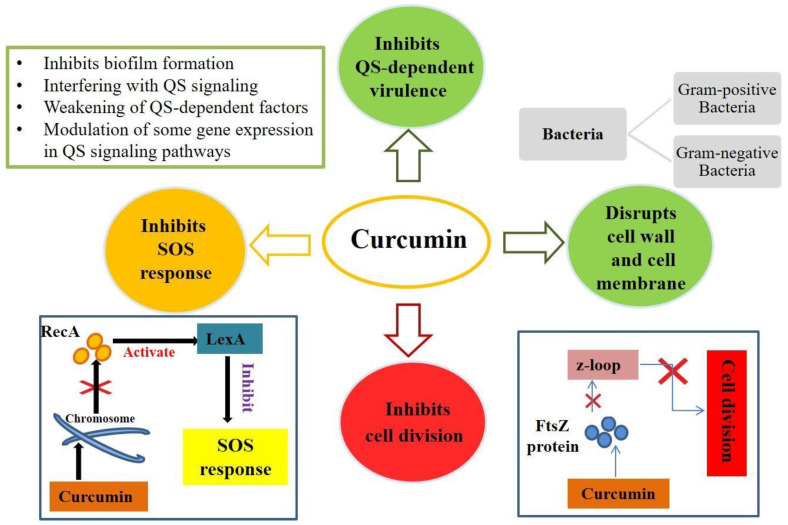
Mechanism of action of curcumin in bacterial growth. Curcumin can inhibit bacterial growth by targeting the bacterial cell membrane, cell wall, protein, DNA, and other cellular structures or by inhibiting bacterial growth through the quorum sensing (QS) system.

The mechanism of action of curcumin is mainly based on the perturbation of the functions of FtsZ, a protein involved in bacterial cell division that is homologous to the cytoskeletal protein tubulin of eukaryotes. In fact, curcumin can inhibit the assembly of FtsZ and the Z loop, which are essential for the cytokinesis of bacteria, resulting in the blockage of bacterial proliferation [42]. In addition, Gram-positive bacteria show greater sensitivity to curcumin than Gram-negative bacteria. Sensitivity to curcumin varies depending on the structure of the cell envelope of the bacteria (Figure 1). In fact, Gram-negative bacteria possess different cellular components that confer this resistance to curcumin [44]; they possess an inner cytoplasmic membrane surrounded by a thin layer of peptidoglycan and an outer membrane containing lipopolysaccharides. The outer membrane serves as a permeability barrier, controls the entry and exit of various substances (such as ions, nutrients, and environmental toxins), and contributes to osmoprotection. Gram-positive bacteria possess an inner plasma membrane and a thicker layer of peptidoglycan but lack a protective outer membrane [45]. This is why Gram-negative bacteria exhibit greater resistance to antimicrobial agents than Gram-positive bacteria [44].

Several studies have been conducted to evaluate the antimicrobial activity of curcumin against different bacterial strains. For example, a study was conducted on MRSA to confirm the antibacterial activity of curcumin on this Gram-positive bacterium [46]. Curcumin has been shown to bind to the cell wall, thus altering bacterial integrity. In addition, it causes damage to the cell membrane: after 8 h of curcumin treatment, transmission electron microscopy (TEM) was used to observe that a cytoplasmic membrane was damaged and, as a result, cell lysis occurred [46]. Another study conducted on *B. subtilis* demonstrated similar results. Following curcumin treatment, the alteration of several proteins—mainly involved in bacterial cell division, cell wall biosynthesis, fatty acid synthesis, and central metabolism—was found. Indeed, fluorescence microscopy showed significant alterations in bacterial cell morphology [47]. A recent study was conducted on a strain of *E. coli* to evaluate the antimicrobial effect of curcumin on this Gram-negative bacterium. At high concentrations of curcumin, a baptismal response similar to apoptosis was recorded. Indeed, at these concentrations, curcumin induced an accumulation of ROS, alteration of membrane potential, depolymerization of the membrane itself, DNA fragmentation, and cell apoptosis [48]. Therefore, curcumin can inhibit bacterial growth by targeting the bacterial cell membrane, cell wall, protein, DNA, and other cellular structures or by inhibiting bacterial growth through the quorum sensing (QS) system (Figure 1). 

To monitor cell density and species complexity in a population, QS (a communication process between microbial cells) uses chemical signals (self-inducers) that are generated and detected. By coordinating collective activities, QS enables bacteria to behave as a coherent community [49]. Curcumin inhibits QS activity through a variety of mechanisms (Figure 1). It can inhibit QS-dependent factors such as exopolysaccharide synthesis in pathogenic strains such as *E. coli*, *P. aeruginosa*, *Proteus mirabilis*, and *S. aureus*. Curcumin has also been shown to diminish several phenotypes associated with QS inhibition such as swimming, clustering, and motility because the swimming and clustering activities of bacteria are major harmful aspects of biofilm development in different bacterial strains. Curcumin can reduce virulence characteristics via the QS system. The signaling molecules of the QS system can modulate virulence factors [50]. Curcumin also inhibits the production of biofilms through the bacterial QS system, not by killing bacteria or destroying the mature biofilm (Figure 1) but by inhibiting the biofilm generation process. Biofilms are bacterial, extracellular, macromolecule-encased clusters of microbial tissues. Biofilm formation is a dynamic process that comprises bacterial adhesion, biofilm growth, and maturation, all of which are regulated by the QS system [51].

## 5. Enhancement of Curcumin Antimicrobial Effects by Photoactivation

**Figure 2:**
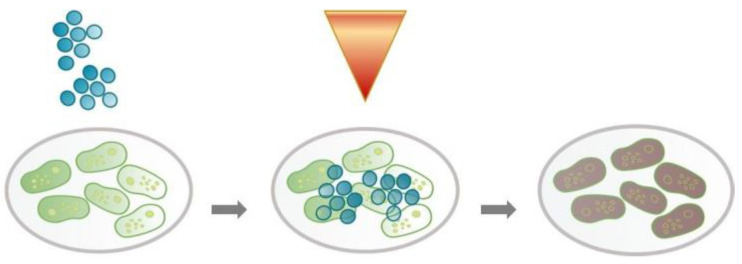
The experimental procedure is graphically depicted. Curcumin interferes with harmful bacterial microorganisms, and the photoactivation of curcumin by means of LEDs enhances the toxic effect on microorganisms.

Photoactivation is based on the local or systemic activation of PS by light with an oxygen source at an appropriate wavelength. The generation of ROS-singlet oxygen and radical species is a result of this activation. Photodynamic therapy is based on a non-thermal photochemical process that requires the presence of a photosensitizing agent (photosensitizer), oxygen, and visible light [52]. The procedure is illustrated in Figure 2.

Recently, researchers have reported interest in enhancing the antimicrobial activity of curcumin by photoexcitation [7,53]. In aqueous solutions, curcumin is photodegradable (light-sensitive) and self-degradable in the dark [54]. However, via illumination (400–500 nm wavelength), curcumin produces ROS, superoxide anions, and hydroxyl radicals, which have lethal effects on bacterial cells, because it is a natural photosensitizer [53].

Photodynamic inactivation (PDI), which has the benefits of safety, environmental protection, and low energy consumption, has been highlighted as a promising strategy against foodborne microorganisms [55,56]. The basic idea of PDI is that particular wavelengths of light activate PS that then form ROS such as superoxide anions, hydroxyl radicals, and hydrogen peroxide, which cause cytotoxic responses that lead to cell death [6]. There have been some attempts to elucidate the mechanism of PDI in foodborne bacteria [57]. ROS may cause DNA damage by targeting guanine nucleotides according to [58], and the authors of [59] reported that curcumin-mediated PDI damaged cytoplasmic DNA and proteins. On the other hand, oxidative bursts of ROS disrupt microorganism homeostasis and the antioxidant enzyme system generates a key cellular defensive network: particularly superoxide dismutase (SOD), catalase (CAT), and glutathione peroxidase (GPx) [60]. Several studies have demonstrated the photodynamic microbial inactivation of some pathogenic bacteria by exploiting an LED light source [61]. At a wavelength of 425 nm, LEDs are able to photoactivate curcumin, as it is a natural photosensitizer and would be very useful in the decontamination of food surfaces. In contrast to traditional light sources, LEDs have several advantages: they are very energy-efficient, cost-effective, durable, and emit little heat. In addition, upon the photoactivation of curcumin, LEDs result in the production of ROS such as superoxide anions, hydroxyl radicals and many others that lead to bacterial cell death. Therefore, the combination of PS and LED illumination would result in the reasonable inactivation of microorganisms on the surface of food. Furthermore, the use of photoactivated curcumin would be a viable option because it is safe, abundant in nature, and does not significantly affect the organoleptic characteristics of food products [53].

The food industry, at present, appears to be very interested in curcumin due to its natural chlorination, antimicrobial and flavoring properties, low cost, and availability. Moreover, because it is a natural photosensitizer, it could be used against a wide range of microorganisms to decontaminate the surface of food. This is the reason why it is considered a “green” and safe alternative compared to common antimicrobial drugs produced by the pharmaceutical industries [7].

Preliminary data obtained by our group [62] showed that curcumin already has a robust effect against some bacterial strains, with low minimal inhibition concentration (MIC) and minimal bactericidal concentration (MBC) values. Moreover, the possible synergistic effect of curcumin combined with photodynamic treatment for 3 h by blue visible light (LED) was evaluated on the same bacteria (two Gram-positive bacteria, *S. aureus* ATCC 29213 and *E. faecalis* ATCC 29212, and one Gram-negative bacterium, *E. coli* ATCC 25922); see Table 2.

The values were confirmed by three independent measures. As the MIC is a discrete value and the overall sensitivity allows for the discrimination of the doubling and halving of the stated value, the error on each measure in the table corresponded to 50%. 

Finally, the antimicrobial effect of curcumin was directly tested by the same research group [63] on samples of fresh fruit (blueberries), which were settled in three containers: 1. blueberries washed with a solution of cyclodextrin (1 mg/mL) (control); 2. blueberries washed with a curcumin and cyclodextrin solution without physical treatment; and 3. blueberries washed with a curcumin and cyclodextrin solution and irradiated daily for 6 h with LED. The three trays, containing a single layer of fruit, were placed in a refrigerator. Each day, at the same time, 3–4 fruits were randomly collected from each tray, transferred to tubes filled with sterile water, and shaken for a few minutes. To assess the presence of microorganisms, the washing water, after fruit extraction with sterile tweezers, was in part directly plated on Brain Heart Infusion Agar (BHA) for bacterial counts and Sabouraud dextrose agar (SAB) to assess the fungal presence and in part filtered through a vacuum filtration unit while changing the unit for each tube, i.e., the filter. Each filter was cut in half: one half was placed on the BHA, and the other half was placed on SAB. All agar plates were incubated at 37 °C for 24–48 h to permit microbial growth. The following day, the plates were compared to assess the microbial load in the washing water. To check their organoleptic characteristics (sight, smell, touch, and taste) the fruits were tasted following extraction from the water. Specifically, volunteer subjects A, B, C and D were asked to taste blueberries and evaluate the appearance of the fruit by sight, smell, touch, and taste; they then assigned a score of 0 if the appearance was altered or 1 if the appearance was normal.

**Figure 3:**
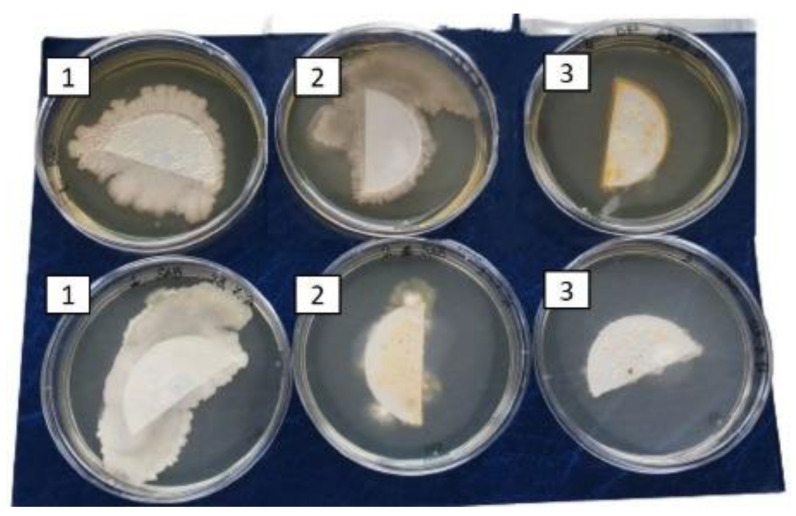
Microbial growth from filter on BHA and SAB plates: 1. Blueberries washed with a cyclodextrin solution (control); 2. blueberries washed with a curcumin and cyclodextrin solution; and 3. blueberries washed with a curcumin and cyclodextrin solution irradiated daily for 6 h with blue LEDs.

**Figure 4:**
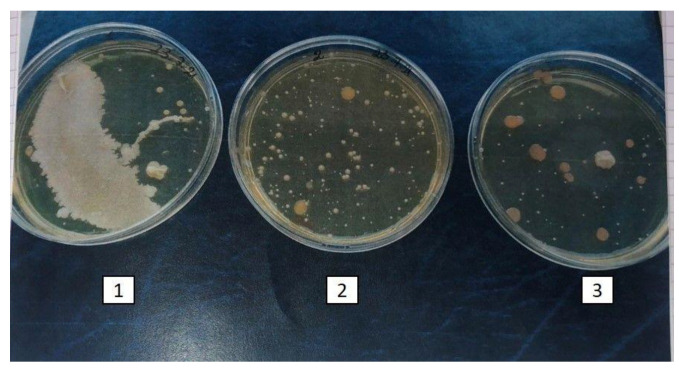
Direct microbial growth on BHA plates: 1. Blueberries washed with cyclodextrin (control); 2. blueberries washed with a curcumin and cyclodextrin solution; and 3. blueberries washed with a curcumin and cyclodextrin solution irradiated daily for 6 h with blue LEDs.

In Figure 3 and Figure 4, a visible reduction in microbial growth, both bacteria or fungi, is evidenced under the third experimental condition, i.e., blueberries washed with a curcumin and cyclodextrin solution and irradiated daily for 6 h with LED.

Table 3 shows the results of the organoleptic qualities analysis of the three samples. Sample 1 did not change because it was not rinsed with curcumin. After being washed with curcumin and not exposed to physical treatments, sample 2 exhibited no organoleptic alterations. Finally, sample 3 exhibited no changes in organoleptic properties after being rinsed with curcumin and exposed to blue LEDs for 6 h.

## 6. Further Considerations on Food Treatment

The most efficient approach to reduce the risks of disease outbreaks and postharvest spoilage is to prevent contamination from primary sources with appropriate sanitation and decontamination methods. Curcumin can have antimicrobial and antibacterial activity, and, because it is a natural photosensitizer, this effect can be enhanced by photodynamic treatment. Various applications have been proposed in recent years, indicating that photodynamic activation can be a very promising sterilization method.

PDI is a technique that relies on the occurrence of non-thermal photophysical and photochemical reactions, requiring light and PS in the presence of oxygen [64]. It works on the principle that PS can be activated in certain wavelengths to produce ROS with strong oxidation to inactivate malignant cells and pathogenic microorganisms [65]. The advantage of PDI is that it does not produce toxic chemicals; the only energy required is the light source, and the possibility of causing microbial resistance is low due to its multi-target nature [66].

Typical light sources are LEDs, lasers, and halogen lamps, and the wavelength of light is a crucial factor for PDI. LEDs have the advantages of low cost, wider emission bands, ease of use, and greater flexibility in irradiation time.

It is known that curcumin may both exhibit antimicrobial properties and produce photodynamic effects to further potentiate its antimicrobial efficacy [67]. Curcumin and illumination were found to synergistically inhibit pineapple slice microorganisms [68].

Other encouraging preliminary results have been obtained for red fruits: following curcumin administration and lighting with LEDs, MIC at a very low concentration of curcumin—i.e., 0.125, <0.0075, and <0.0037 mg/mL against *E. coli*, *S. aureus*, and *E. faecalis*, respectively—were found (see Table 2).

Such data showed that curcumin combined with photodynamic treatment by blue visible light (LED) led to impressive variations in MIC (one or even two orders of magnitude) in antibacterial activities against Gram-positive bacteria. 

Note that the minimal concentration of curcumin required for *S. aureus* (MIC corresponding to 20.3 μM with LED irradiation) is far lower than that reported in Corrêa et al. [10] on apples (80 μM) and beef, chicken, and pork meat (40 μM).

In addition, curcumin treatment with and without LED photoactivation was used on a fruit sample (blueberries), and microbiological and organoleptic (sight, smell, touch, and taste) investigations showed that it was effective without altering taste (see Table 3).

Microbial decontamination via curcumin photoinactivation has been successfully applied to oysters [69], showing that the food matrix was minimally oxidated [70] and could therefore maintain its organoleptic properties. The same procedure was extended to other seafood [71].

As far as fruit is concerned, experiments were performed on dates and showed that small curcumin concentrations (few nM) could greatly prolong the shelf-life period [72]. Investigations into apples have also been reported [73].

Very recently, curcumin’s natural properties and their enhancement by photodynamical inactivation have been tested on various fungal strains [74,75] such as *Candida albicans*, *Aspergillum* (*niger* and *flavus*), and aflatoxins.

Regarding viruses, the same approach was tested against feline calicivirus (FCV) and murine norovirus (MNV) with satisfactory results [76].

In general, all the above-mentioned studies show that the effectiveness of PDI is strongly dependent on the bacterial strain, e.g. particular Gram-negative strains are far less affected. Unfortunately, very few researchers have investigated why the mechanism of the antibacterial activity of curcumin seems to differ depending on the bacterial strain being studied. Tyagi et al. [32] showed that curcumin attacks both Gram-positive and Gram-negative bacteria in a similar manner by causing membrane permeabilization. Aurum et al. [53] showed that LED illumination alone had negligible antibacterial effects but photoactivated curcumin exhibited strong bactericidal activities against *E. coli*. In our study, a slight minor efficacy was evidenced in *E. coli*, and this result could be attributable to the different bacteria inocula, curcumin formulations, and experimental conditions we used. A study by Shlar et al. [44] provided strong evidence that the mode of biological activity of curcumin depends on the properties of the delivery system.

These variations are possible due to the different cell wall compositions of Gram-positive and Gram-negative bacteria. Gram-positive bacteria are easily killed due to their porous structure and easy penetration of PS. On the other hand, Gram-negative bacteria are not easily killed due to their outer membrane that gradually block the activity of curcumin.

Similar results have been published by other authors since the first studies [64,77,78,79], and it has been speculated that different results have resulted from different membrane structures. Nevertheless, when curcumin is delivered by nanovectors (which are expected to facilitate membrane crossing [80,81]), the outer membrane of Gram-negative bacteria protects them from curcumin diffusion and photosensitization.

Curcumin-loaded nanoparticles and chitosan-modified curcumin-loaded nanoparticles were prepared by Agel et al. to quantify their antibacterial photodynamic effects against *Staphylococcus saprophyticus* subsp. *bovis* and *E. coli* DH5 alpha. The researchers demonstrated that neither irradiation alone nor curcumin in the absence of light could lead to significant growth reductions, confirming the photodynamic effect of curcumin. The increased adherence of the chitosan-modified nanoparticles to bacteria and structural damage upon photodynamic treatment were evident and confirmed the results of in vitro studies. Moreover, curcumin-loaded nanoparticles in combination with LED irradiation resulted in a *S. saprophyticus* survival rate of less than 0.0001% (>6.2 log10 reduction). In the presence of nanoparticles loaded with chitosan-modified curcumin in combination with light, the efficacy was greater, with a survival rate of 0.0000045%. In contrast, in the presence of *E. coli*, the photoactivated curcumin-loaded nanoparticles showed a survival rate of 0.13% (CFU only ~2.9 log10) and an increase in antibacterial efficacy with the curcumin-loaded nanoparticles modified with chitosan with LED, with an up to 5.9 log10 reduction in CFU (0.00013% survival) [80].

## 7. Photoactivated Curcumin as ‘Green’ Tool for Food Preservation: Future Perspective

In conclusion, this review discusses the emerging role of curcumin, alone or together with photodynamic light, in enhancing antimicrobial and antibacterial activity and finding effective strategies to preserve food. The preliminary results of the proposed research can be promising in the fight against harmful microorganisms in fruits, potentially improving food quality.

The development of innovative systems based on natural products and physical methods (such as PDI) in the food industry is considered a promising future perspective for alternative “green” techniques in food preservation.

Before proposing applications at the industrial scale, this technique still requires the optimization of the typical parameters that must be considered in experiments with LEDs, i.e., the light wavelength (nm), the emission spectra, the irradiance (J∙cm^−2^), and LED potency (W).

Along with light sources, the temperature and acidity of food affect the efficacy of PS and antibacterial effects, so attention should be paid to all phases of food treatment and shelf life. However, due to its low cost and simple applicability, photoactivated curcumin is a promising and biofriendly technique for preserving food by bacterial contamination.

## Figures and Tables

**Table 1:** Some of the main bacteria contaminant of food.

Bacteria	Gram	Contaminate Food	Health Damage Mechanism	Antibiotic Resistance
*E. faecalis*	+	Cheeses, fermented sausages, andready-to-eat food.	Enterococcal surface proteins, hyaluronidase, gelatinase, and biofilm production [14].	The most prevalent antibiotic resistances were found to be tetracycline, minocycline, erythromycin kanamycin, and chloramphenicol [15].
*S. aureus*	+	Fruits, meat, egg products, milk and its derivatives, salads, and baked goods such as pastries and cream-filled desserts.	Staphylococcal enterotoxins (SEs) causing Staphylococcal foodborne disease (SFD) [16,17].	Foodborne strain methicillin-resistant *S. aureus* (MRSA) with the highest resistance rate to penicillin G, ampicillin, and erythromycin [18,19].
*E. coli* (specifically the enterohemorrhagic serogroup O157:H7)	-	Dairy products, delicatessen products, salads, spices, cream cakes, and fresh fruit and vegetables.	Shiga toxins, pathogenicity island products, and F-like plasmid pO157 products.	Colistin had the lowest prevalence (0.8%) and amoxicillin had the highest (70.5%), a recent and significant increase in ciprofloxacin resistance [20,21].

**Table 2:** MIC and MBC of curcumin alone tested against *E. faecalis*, *S. aureus,* and *E. coli* with or without photodynamic treatment on 3 different bacterial strains.

Bacteria	MIC (mg/mL) No LED	MIC (mg/mL) 3 h LED	MBC (mg/mL) No LED	MBC (mg/mL) 3 h LED
*E. faecalis*	0.125 ± 0.063	0.0037 ± 0.0019	>0.25	0.0037 ± 0.0019
*S. aureus*	0.06 ± 0.03	0.0075 ± 0.063	>0.25	0.0075 ± 0.0038
*E.coli*	0.125 ± 0.063	0.125 ± 0.063	>0.25	>0.25

**Table 3:** Organoleptic aspects of three samples: 1. Blueberries washed with water (control); 2. blueberries subjected to washing with curcumin; and 3. blueberries subjected to washing with curcumin irradiated for 6 h with blue LEDs. V: sight; O: olfaction; T: touch; G: taste; A–D signify subjects who ate blueberries to evaluate their organoleptic characteristics. 0 = alteration of the characteristic; 1 = no alteration of the characteristic.

	1	2	3
Subject	V/O/T/G	V/O/T/G	V/O/T/G
A	1/1/1/1	1/1/1/1	1/1/1/1
B	1/1/1/1	1/1/1/1	1/1/1/1
C	1/1/1/1	1/1/1/1	1/1/1/1
D	1/1/1/1	1/1/1/1	1/1/1/1

## Data Availability

The presented data are available on request from the corresponding author.
